# Monitoring effect of nickel, copper, and zinc on growth and photosynthetic pigments of *Spirulina platensis* with suitability investigation in Idku Lake

**DOI:** 10.1007/s11356-022-21328-1

**Published:** 2022-06-15

**Authors:** Mona Kaamoush, Nagwa El-Agawany, Hamida El Salhin, Ahmed El-Zeiny

**Affiliations:** 1 Environmental Protection and Crises Management Department, Simulator Complex, Arab Academy for Science, Technology and Maritime Transport, Alexandria, Egypt; 2 Botany and Microbiology Department, Faculty of Science, Alexandria University, Alexandria, Egypt; 3 Environmental Studies Department, National Authority for Remote Sensing and Space Sciences (NARSS), Cairo, Egypt

**Keywords:** Nickel, Copper, Zinc, Pollution, Remote sensing, Spatial modeling, *Spirulina platensis*

## Abstract

Owing to the increase of pollutant sources in oceans, seas, and lakes, there is an expected effect on growth and metabolism of planktonic algae which are considered primary producers in the ecosystem. Therefore, it becomes urgent to carry out laboratory studies to test to what extent these pollutants can affect the growth of algae which is necessary as a food for marine fishes. *Spirulina* is considered the most important algal species due to its high nutritional value for humans and animals. Therefore, this work investigated the effect of different concentrations of Ni^2+^, Zn^2+^, and Cu^2+^ metal ion pollutants on growth of the blue-green alga *Spirulina platensis*. EC50 was identified to be around 2 mg/l for the three heavy metals. The suitability of Idku Lake for *Spirulina platensis* growth was investigated using multi-criteria spatial modeling integrated with remotely sensed data processing. Spatial distribution maps of turbidity, water nutrients, and phytoplankton were the input criteria used to assess Idku Lake’s suitability. The results obtained proved that low concentrations of the tested heavy metals stimulated growth and pigment fractions (chlorophyll a, carotenoids, and total phycobilins content) but to different degrees. The inhibitory effect was more prominent in the case of copper ions than zinc and nickel ions with all concentrations used. The overall suitability map of *Spirulina platensis* in Idku Lake showed that the whole lake is suitable for growth and proliferation except for the northwestern corner due to the high salinity levels. The present paper helps to understand the behavior of algae responding to environmental pollution, which supports environmental planners with the necessary baseline for investigating the fate of pollutants and the potential risk.

## Introduction

Aquatic ecosystems perform numerous important environmental functions. For instance, they purify water, attenuate floods, recycle nutrients, recharge groundwater, and also afford habitats for wildlife. The aquatic environment is one of the significant media that should be protected from pollution. Approximately 1500 substances have been identified as pollutants in aquatic ecosystems, for instance, acids and alkalis, anions (e.g. sulfide, sulfite cyanide) detergents, municipal sewage and farm manures, wastes of food processing, gases (e.g. chlorine, ammonia), heat, heavy metals (e.g., lead, cadmium), nutrients particularly phosphates and nitrates, oil and oil dispersants, organic toxic wastes (e.g., formaldehydes, phenols), pathogens, pesticides, polychlorinated biphenyls, and radionuclide. Whether or not a compound will exert an effect on an organism or a community will depend on the concentration of that compound and the time of exposure to the compound. The effect of a pollutant on a target organism may be either acute or chronic (Saunder 1976).

The essential heavy metals (e.g., Cu, Fe, Mn, Se, Zn, etc.) are important for biochemical and physiological functions in living organisms (Tchounwou et al. 2012). Copper, for instance, is one of the valuable metals in many industrial processes, e.g., textile, painting, metal finishing, electroplating, and plastics (Al-Saydeh et al. 2017). In addition to copper, its effluents contain other elements, in lower concentrations, such as iron, zinc, nickel, etc. These metals may also have an undesirable impact on living organisms and the environment. Accordingly, copper-contaminated effluents have to be treated before discharge into natural water bodies. The common methods developed by microorganisms for removing from the environment include biotransformation, bioaccumulation, biomineralization, and biosorption (Ayangbenro and Babalola 2017; Cepoi et al. 2020).

Algal communities have been utilized in tests of toxicity for environmental monitoring of heavy metal pollution and can be used in determining general water quality and growth-limiting nutrients. Likewise, they can be used in the removal of metals from contaminated wastewater (Terry and Stone 2002). Marine phytoplankton is vital for the normal functioning of ecosystems since it forms the basis of the marine food chain. The disturbance to this component is likely to exert influences on higher trophic levels, which may be due to the release and accumulation of toxic compounds (Arora et al. 2002).

The genus *Spirulina* (Arthrospira) is considered one of the microalgae having commercial importance*.* Its biomass is extensively applicable in biofertilizers, feed, cosmetics, biofuels, food, and biomaterials. *Spirulina* is distinguished from other foods, since it grows in extreme environments. It grows in very alkaline water (> pH 9) where no other plant can grow, i.e., it does not require pesticides or herbicides. This means that *Spirulina* forming in the long term is the best and softest method to produce healthy foods without destroying the environment (Costa et al. 2019). *Spirulina* is a healthy whole food alga where it can supply the human body with the majority of essential elements and vitamins. The covering cell wall is not cellulose but a thin membrane that is composed of complex sugars which dissolve directly in the digestive juice of the stomach. *Spirulina* is considered a good food to sustain health and it is a good aid to regain health. Its metabolic products are quite sufficient to cover all these two items. It gets its own sun protective defense from the higher β-carotene production in the intensive sun. The hotter and stronger the sun shines, the better the quality of the product. Therefore, for *Spirulina*, nature itself is the best physician; it has a high nutritional value associated with its vitamin, mineral, and fatty acid content which are important for different biological activities, and easy digestibility (Lupatini et al. 2017; Trivedi et al. 2015). *Spirulina* contains several minerals such as magnesium, calcium, iron, and phosphorus. It represents a main source of iron as it contains 20 times more iron than a wheat gram (Cuellar-Bermudez et al. 2015; Ravindran et al. 2016).

Photosynthetic pigments in *Spirulina* are very important in different fields. Chlorophyll is one of the important pigments in *Spirulina*; it plays an important role in light capturing and contributes to anti-oxidants activities in algae, also it is used as a colorant in food and different pharmaceutical industries (Yuliani et al. 2019). Carotenoids have a valuable effect to influence the signaling and regulation of many biological pathways. Moreover, they have significant antioxidant activities (Ranga et al. 2018). In plants, carotenoids are excellent scavengers of singlet oxygen; thus, they protect cellular components, such as chlorophylls, lipids, proteins, and DNA, from oxidative damage in cells. Carotenoids are very important to human health; they enhance the immune system function and play important roles in the protection of different diseases (Raposo et al. 2015; Wang et al. 2002). Carotenoids, chlorophyll derivatives, and phycobilins are among the antioxidants with the highest activity in *Spirulina sp.* (Jaime et al. 2005).

Adding small amounts of microalgae biomass is valuable for the physiology and the immune response of animals. It also boosts antiviral resistance, antibacterial, and intestinal function. These factors result in growth promotion, weight control, improved feed conversion, and reproductive performance (Harel et al. 2007).

*Spirulina platensis* is an un-branched filamentous blue-green alga attaining the size of 0.5 mm in length and occurs naturally in highly alkaline lakes. *Spirulina sp.* is present in different Egyptian lakes especially Lake Mariut and Lake Idku. Lake Mariut is extensively polluted as it suffered from serious ecological damage due to industrialization and modern agricultural systems (Abd El-Hamid et al. 2021). As a result of the extensive nutrients input received from Alexandria city, the enclosed nature of the lake and the water shallowness, heavy algal blooms, and domination of plankton occurred particularly *Spirulina sp*. On the other hand, Lake Idku is characterized by high nutrient concentrations of NO_2_-N (5.4 μg/l), NH_4_-N (4.5 μg/l), PO_4_-P (7.8 μg/l), and SiO_2_-Si (97.7 μg/l) approximately, as well as a high level of dissolved oxygen (8.3 mg/l). This is mostly attributed to the excessive amounts wastewater discharged into the lake (Khairy et al. 2015). This alga used in this research was kindly provided by Prof. Yahia Azab, Algal Culture Collection of El-Mansoura University, Egypt. *Spirulina platensis* was grown in *Spirulina* medium (Zarrouk 1966).

Applications of remote sensing and geographic information system are widely used in environmental assessment studies. Extracting environmental variables from remotely sensed imagery is important to facilitate studying regional and inaccessible areas which cannot be investigated using traditional methods. The integration of remote sensing with spatial modeling helps for environmental site selection studies (Effat and El-Zeiny 2017; 2020; El-Zeiny and Effat 2019; Al-Shaibah et al. 2021).

Recently, several research studies in Egypt have been carried out to investigate different environmental components associated with biological factors such as *Dunaliella* algae, mosquito, and wild plant habitats (El Agawany et al. 2021; El-Amier et al. 2021; Nagy et al. 2021).

The spatial distribution maps using geostatistical analyses represent one of the most common applications of geographic information systems. These analyses help to generate continuous surfaces by predicting values based on real measurements (El-Zeiny et al. 2019). Generation of geographic databases with all available resources and potential hazards is important for decision-making process Therefore, present study aims to assess the effects of different heavy metals concentrations on the growth and photosynthetic pigmentation of *Spirulina platensis.* Furthermore, the suitability of Idku Lake for the proliferation of *Spirulina platensis* is tested seeking for identifying the most suitable part of the lake using the cartographic modeling techniques.

The new knowledge added from this paper can be summarized as scientific and economic-environmental knowledge. The scientific sector is the integration between lab analyses and spatial techniques to test the algae response to different levels of heavy metals and the suitability of Idku Lake for proliferation which represents a practical application to the lab experiment in a real case study. The economic-environmental knowledge can be expressed in resolving a severe environmental problem through Spirulina algae indicating biological treatment with the proliferation of the algae with an economic value.

## Materials and methods

### I-Biological material

In the present study, the tested biological material was the axenic filamentous cyanobacterial alga *Spirulina platensis*. This alga was kindly provided by Prof. Yahia Azab, Algal Culture Collection of El-Mansoura University, Egypt. *Spirulina platensis* was grown in *Spirulina* medium (Zarrouk 1966) with specific component as shown in Table 1.

**Table 1:** Composition of *Spirulina* medium (Zarrouk, 1966)

Macronutrient stock solution		Micronutrient stock solution			
Stock solution a	Quantity (g.)	Stock solution b	Quantity (g.)	Stock solution c	Quantity (g.)
1- NaCl	1.0	1- NH4NO3	0.023 g	1- H3BO3	2.82 0 g
2- MgSO_4_. 7H_2_O	0.2	2-K_2_Cr_2_ (SO4)_2_. 27 H_2_O	0.096 g	2- MnCl_2_. 4H_2_O	1.810 g
3- CaCl_2_. 2 H_2_O	0.04	3- NiSO_4_. 7H_2_O	0.044 g	3- ZnSO_4_. 7H_2_O	0.222 g
4- FeSO_4_.7 H_2_O	0.01	4-Na_2_SO_4_. 7H_2_O	0.018 g	4- CuSO_4_. 5H_2_O	0.077 g
5- Na-EDTA*	0.08	5- Ti (SO4)_3_	0.040 g	5- MoO_3_	0.015 g
6- K_2_HPO_4_	0.5	6-Co(NO3)6H_2_O	0.044 g	6- Distilled H_2_O	1000 ML
7- NaNO_3_	2.5	7- Distilled H_2_O	1000 ml		
8- K_2_SO_4_	1.0				
9- NaHCO_3_	16.8				
10- Distilled H_2_O	1000 ML				

One ml from stock solution b + 1.0 ml from stock solution c were added to each 1000 ml of solution a

### I-i-Growth measurements by optical density

The growth of the investigated alga (*Spirulina platensis*) was determined by optical density which was determined at 488 nm and compared to calibration standard curve. The optical density was calculated according to the following equation (Robert 1979):

$$\mathrm{Optical}\;\mathrm{density}\;\left(\mathrm O.\mathrm D\right)=\log\;{\mathrm I}_0/\mathrm I$$

where: I is the transmittance of sample. I_O_ is the transmittance of blank adjusted to read 100%.

However, the following equations were also used for calculation of the mean growth rate, growth rate, and logistic growth model from the obtained O.D (biomass).

### I-ii-Growth rate

The growth rate is calculated from the equation:

$$\mathrm{Growth}\;\mathrm{rate}=\left(\ln\;{\mathrm B}_1-\ln\;{\mathrm B}_0\right)/\left({\mathrm t}_1-{\mathrm t}_0\right)$$

where: B_1_ is the optical density at time t_1_. B_0_ is the optical density at time t_2_. t_1_ is the time at the beginning of the experiment. t_2_ is the time at the end of the experiment.

### Mean growth rate

The mean growth rate is calculated from the equation:

$$\mathrm{Mean}\;\mathrm{growth}\;\mathrm{rate}=\left(\ln\;{\mathrm B}_{\mathrm t}-\mathrm{Ln}\;{\mathrm B}_0\right)/\mathrm t$$

where: B_t_ is the optical density at time t. B_0_ is the optical density at the beginning of the experiment.

### I-iii-Logistic growth model

The maximum growth rate (r max) is calculated at the exponential phase of growth from the equation of growth rate. After that, r max was used to calculate the whole sigmoid growth where growth rate (r) at any period of time:

$$\mathrm{r}=\mathrm{rmax}\left(1-\mathrm{B}/\mathrm{K}\right)$$

where: B is the optical density. K is the carrying capacity (maximum yield).

Then r max, K, and X_0_ (initial biomass) were used to calculate change in biomass using sigmoidal growth model.

### II- Estimation of photosynthetic pigments

#### i- *Chlorophyll* “*a*”

The spectrophotometric method is the easier method for measuring chlorophyll “a” and the results calculated according to the trichromatic equation of Jeffrey and Humphrey (1975):

$$\mathrm{Chlorophyll}\;"\mathrm a"\left({\mathrm{mg}.1}^{-1}\right)=\left(11.85\;{\mathrm E}_{664}-1.54\;{\mathrm E}_{647}-0.08\;{\mathrm E}_{630}\right)\mathrm{Va}/\mathrm{Vs}$$

where: Va is the volume of the extract. Vs is the volume of the algal suspension.

#### ii- *Carotenoids*

The spectrophotometer method suggested by Jensen and Liaaen (1959) was used in this investigation. The concentration of carotenoids was calculated using the following equation:

$$\mathrm{C}={\mathrm{D}}_{\mathrm{x}}{\mathrm{V}}_{\mathrm{x}}{\mathrm{F}}_{\mathrm{x}}10/2500$$

where: C is the concentration of carotenoids (mg.l^−1^). D is the absorbance at 450 nm. F is the dilution factor. V is the volume of algal suspension. 2500 is an average of extinction coefficient.

#### iii- *Phycobilins*

Phycobilins were determined according to the method described by Bennett and Bogorad (1973). The concentrations of phycocyanin, allophycocyanin, and phycoerythrin in crude extracts were calculated as mg/l by using the following equations:

$$\mathrm{Phycocyanin}\;\left(\mathrm{pc}\right)=\frac{A_{615}-0.474\;A_{652}}{5.34}$$

$$\mathrm{Allophycocyanin}\;(\mathrm{Apc})=\frac{A_{652}-0.208\;A_{615}}{5.09}$$

$$\mathrm{Phycoerythrin}\;(\mathrm{PE})=\frac{A_{526}-2.41\left(pc\right)-o.849\;\left(Apc\right)}{6.62}$$

### III-Preparation of different concentrations of nickel, copper, and zinc

Three heavy metals, namely nickel, copper, and zinc, were selected during the present study based on their abundance in wastewater as well as their effect on the receiving aquatic ecosystems. Stock solutions of the selected heavy metals were prepared from their salts in double distilled water and sterilized by filtration through 0.2 µm nitrocellulose membranes. The different concentrations of the used selective heavy metals in the metal bioassays were prepared by dilution with double distilled water. As stated by Wong and Pak (1992), an initial experiment using a varied range of metal solutions (NiCl_2_, CuSO_4_.5H_2_O, and ZnCl_2_) was achieved to identify the appropriate concentrations of these metal salts that could be tolerated by the investigated alga. Selections of a series of concentrations were based on the response of the studied alga which had a slightly or noticeable impacts on its growth, and also to exclude the non-effective and directly deadly concentrations on the experimented alga. Consequently, the following concentrations were selected: 1.0, 1.5, 2.0, 2.5, and 3.0 mg/l respectively for each heavy metal.

### IV-Suitability of Idku Lake for Spirulina platensis and satellite imagery analyses

**Fig. 1:**
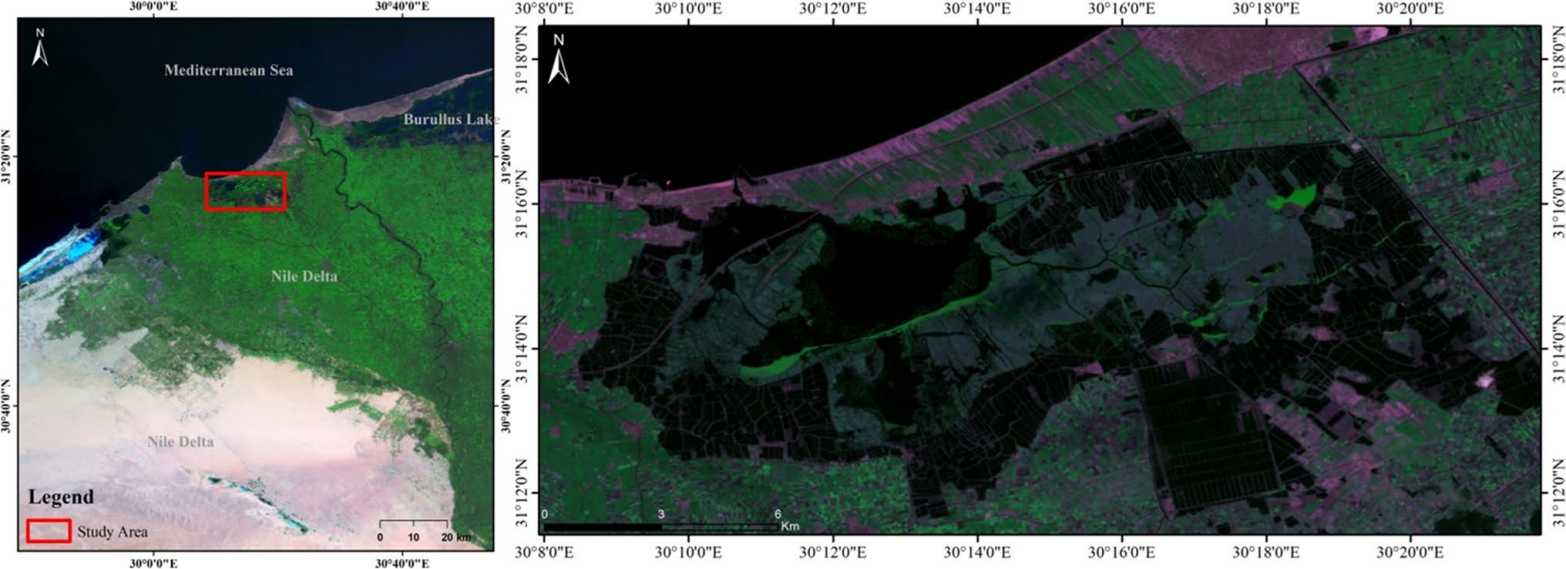
Location map of Idku Lake

The suitability of Idku Lake was evaluated for proliferation of *Spirulina platensis* using geospatial techniques. Idku Lake was chosen as one of the highly polluted Egyptian lakes which receives saline water from the Mediterranean Sea through different water inlets, namely “Boughaz” and receives freshwater from different fresh drains distributed along with the southern parts of the Lake (Fig. 1).

**Fig. 2:**
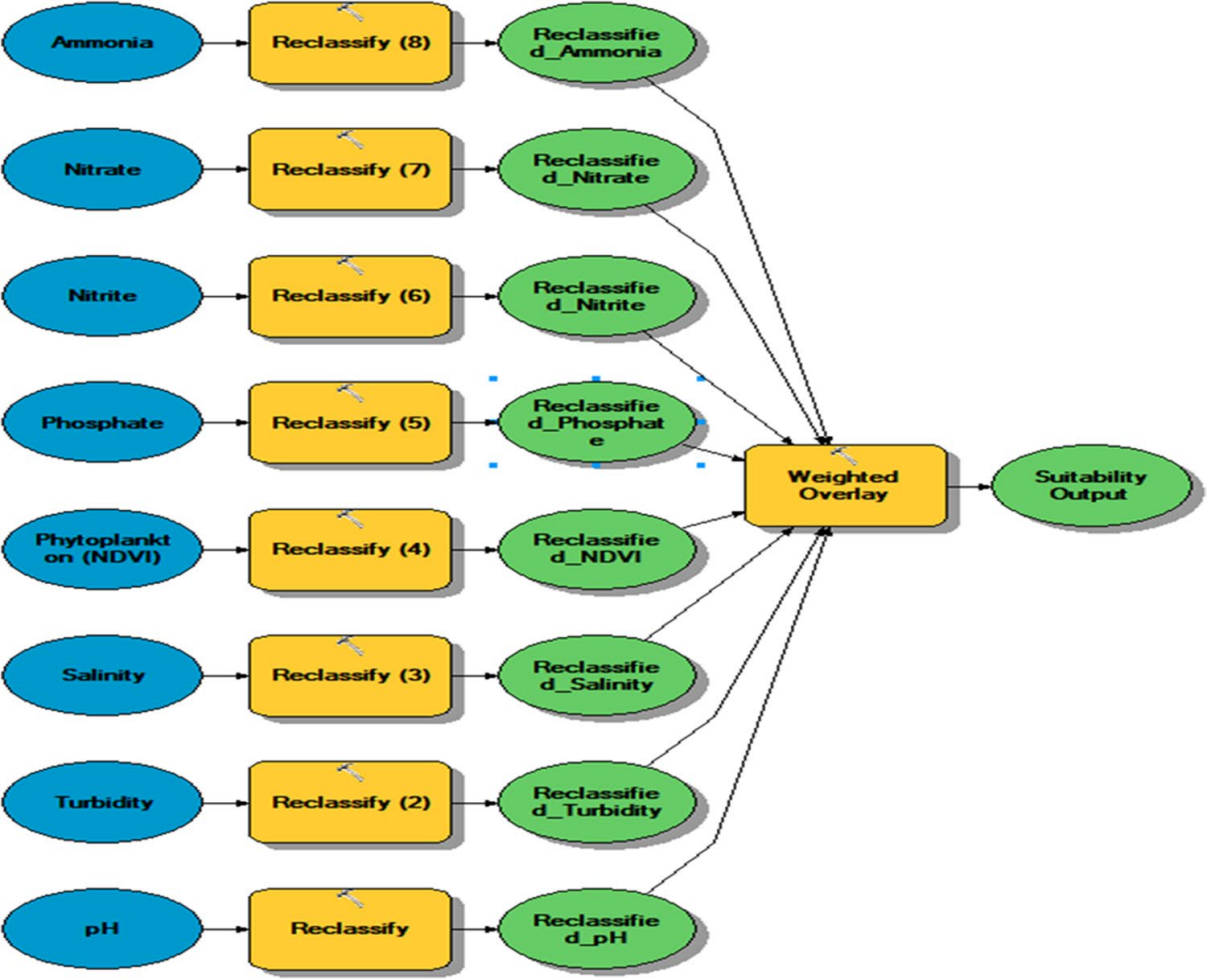
Weighted overlay model for *Spirulina platensis* suitability of Idku Lake

On basis of the optimal water quality parameters of *Spirulina platensis*, the suitability maps were produced. Suitability is increased with the elevation in alkalinity, phosphate, nitrate, nitrite, phosphate, ammonia, and with the decline of TDS and turbidity. A recent multispectral Landsat image dated 17 March 2021 was preprocessed to calibrate the data to produce the NDVI which was used for the identification of phytoplankton content of the lake; higher levels of NDVI indicate more concentration of chlorophyll. Radiometric correction, dark object subtraction, and spatial subset were the preprocessing steps followed to calibrate the data and convert it into reflectance for further processing including indices calculation. NDVI was calculated from the red and NIR bands: NDVI = (NIR − Red)/(NIR + Red). Thus, suitability map for each single parameter was generated and converted into a “raster” layer. These layers were reclassified and inserted into a weighted overlay model to identify the most suitable part of the lake considering all investigated variables (Fig. 2).

## Results and discussions

In our country, we have little information about the ambient concentrations of heavy metals. It is not correct to rely on the data present owing to the presence of many different polluted regions. Heavy metals contamination in water has a seriously harmful effect on living organisms (i.e., Flora and Fauna). Thus, the system of the present work was partitioned into two parts:

### Part I

**Fig. 3:**
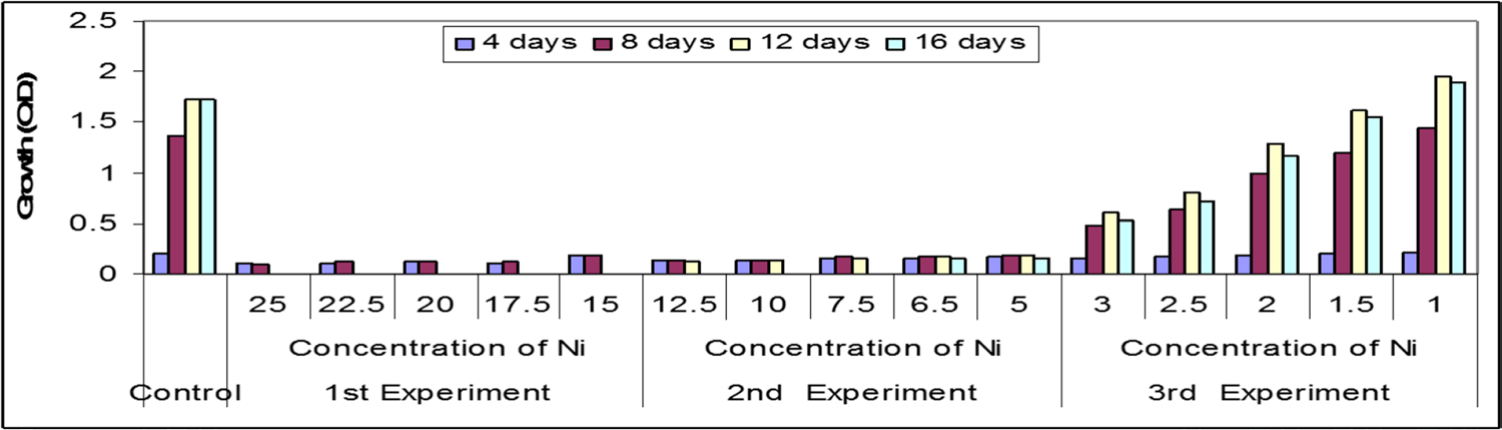
Preliminary experiments of the effect of different concentrations of Ni.^2+^ (mg/l) on growth (O.D.) of *Spirulina platensis*

**Fig. 4:**
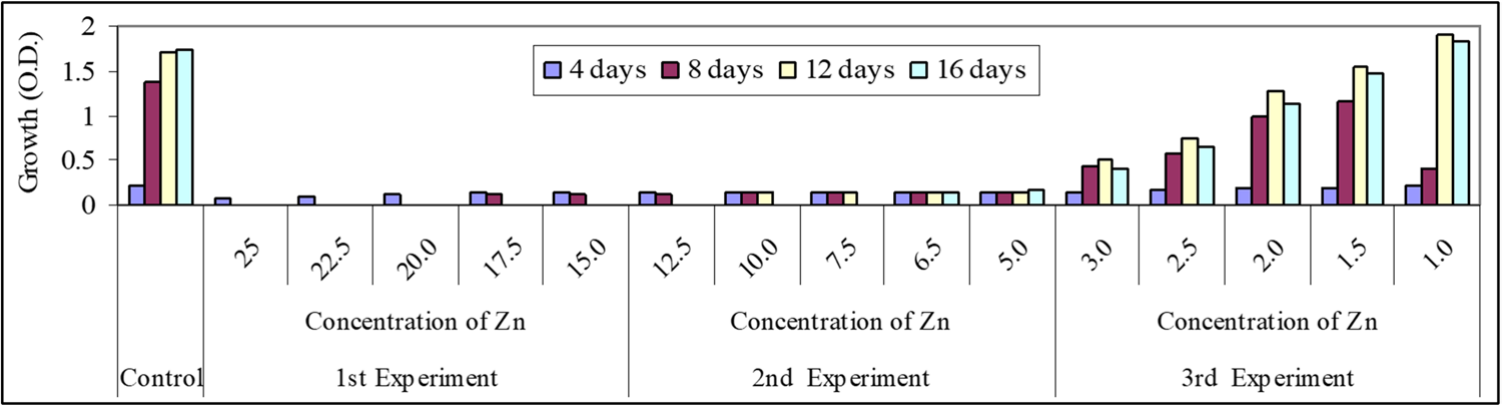
Preliminary experiments of the effect of different concentrations of Zn.^2+^ (mg/l) on growth (O.D.) of *Spirulina platensis*

**Fig. 5:**
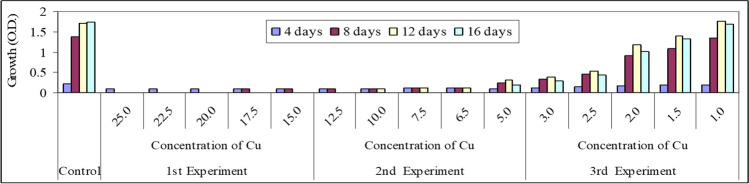
Preliminary experiments of the effect of different concentrations of Cu.^2+^ (mg/l) on growth (O.D.) of *Spirulina platensis*

In this part, preliminary experiments were conducted on the effect of the three selected heavy metals Ni^2+^, Zn^2+^, and Cu^2+^ on the growth of *Spirulina platensis* in order to choose the suitable concentrations that could be safely tested (Figs. 3, 4, and 5). The organism was firstly cultured under selected concentrations of these heavy metals, namely 25.0, 22.5, 20.0, 17.5, and 15.0 mg/l. The obtained results cleared that the organism differently responded under the stress effect of these heavy metal ions and died after 4 days of culturing. The determinable effects were clearer in case of Cu than in the other two heavy metal ions. Therefore, the foregoing experiments were carried out under fewer concentrations than in the case of the first experiment. These concentrations were 12.5, 10.0, 7.5, 6.5, and 5.0 mg/l.

These results go with those obtained by Nalimova et al. (2005) who found that the lethal effect of Cu and Zn on *Spirulina platensis* was 5 and 8.8 mg/l respectively. The results obtained cleared that a sudden drop in growth of the tested organism was recorded at the 8th day of culturing. The organism appeared pale in color and dropped in the bottom of the culturing flasks especially at Cu and Zn elements. Finally, a third experiment was carried out at concentrations of 3.0, 2.5, 2.0, 1.5, and 1.0 mg/l for Ni, Zn, and Cu ions. Under these selected concentrations, *S. platensis* remained alive but with different rates of growth. The results cleared also that the effective concentration (EC50) of Ni, Zn, and Cu was recorded nearly at a concentration 2.0 mg/l after 8 days of culturing. Similar results obtained by Nalimova et al. (2005) who found that the addition of 2.55 mg/l Cu^2+^ on the second day of culturing increased culture growth and when Cu^2+^ levels were increased to 3.8 mg/l, the growth rate and culture productivity decreased on the fourth day. Consequently in this work, five concentrations were chosen (two concentrations below and two higher than 2.0 mg/l), i.e., l.0, 1.5, 2.0, 2.5, and 3.0 mg/l for each element beside control.

### Part II

This part was concerned with the effect of the five different concentrations of Ni, Zn, and Cu that have been chosen from part I on growth and content of some related metabolic compounds of *S. platensis* cells.

#### Effect of different concentrations of Ni^2+^, Zn^2+^, and Cu.^2+^ ions on growth of Spirulina platensis measured as optical density

**Fig. 6:**
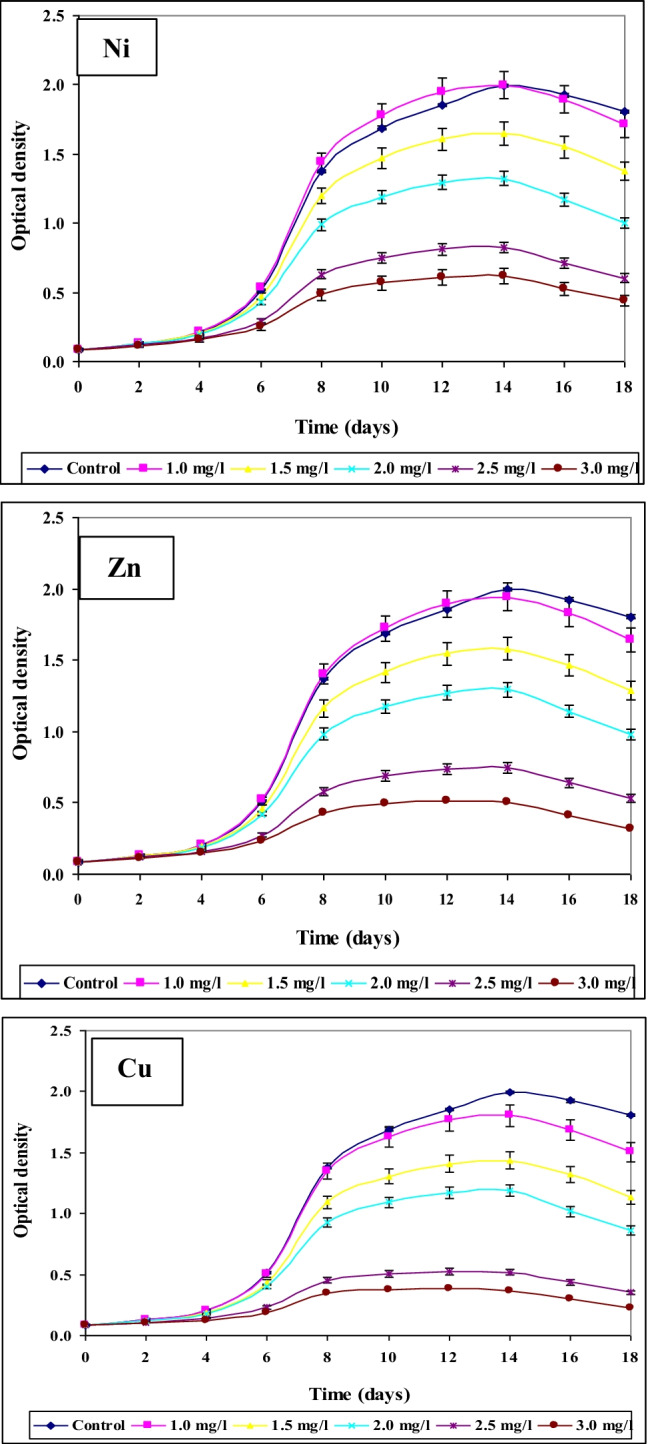
Effect of different concentrations of Ni^2+^, Zn^2+^, and Cu.^2+^ on growth of *Spirulina platensis* cultured for 18 days based on optical density

**Fig. 7:**
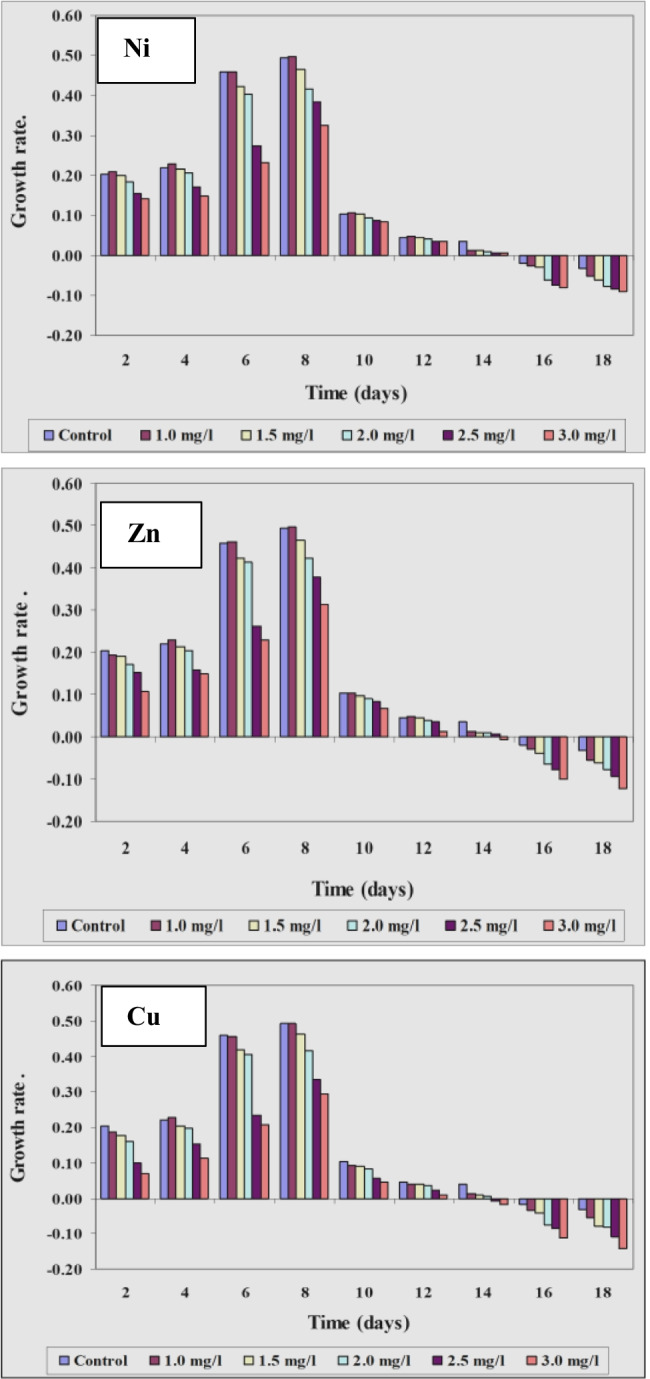
Effect of different Ni^2+^, Zn.^2+^, and Cu concentrations on growth rate of *Spirulina platensis* cultured for 18 days based on optical density

**Fig. 8:**
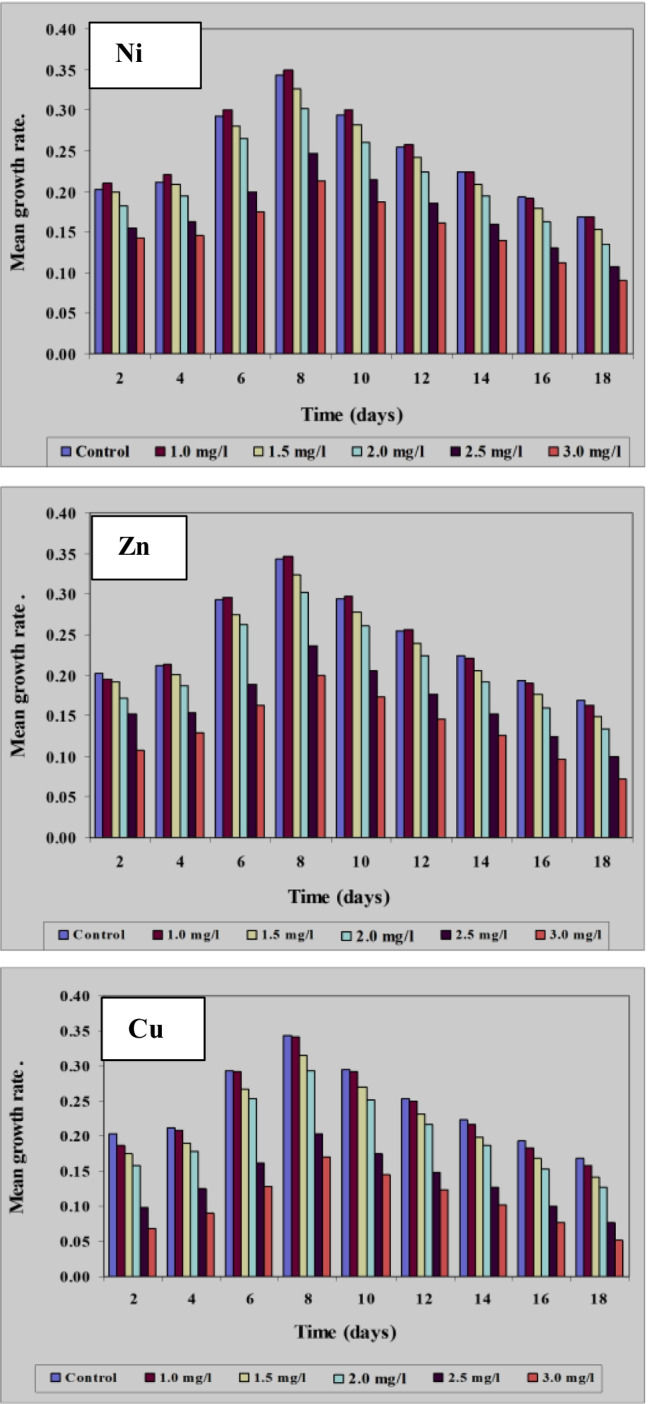
Effect of different Ni^2+^, Zn^2+^, and Cu.^2+^ concentrations on mean growth rate of *Spirulina platensis* cultured for 18 days based on optical density

Risk assessment is a continually evolving process as important information on different contaminants especially heavy metals, the health effects involved, and their occurrence in food are all factors that should be continuously measured and monitored. In correlation with the results obtained by using optical density (O.D.), parameters of growth of *Spirulina platensis* at the control and under the effect of the different concentrations of Ni, Zn, and Cu were recorded in Figs. 6, 7, and 8. With regard to the growth parameters based on O.D. (growth rate and mean growth rate) cleared that maximum growth rate and maximum mean growth rate were obtained on the 8th day of culturing for all the concentrations of these three tested elements but with different values, these results coincide with those obtained by El-Maghrabi (1997). For Ni, the maximum growth rate at the 8th day of culturing reached 0.498, 0.466, 0.415, 0.385, and 0.325 mg/l at concentrations 1.0, 1.5, 2.0, 2.5, and 3.0 mg/l, respectively. For Zn and under the same tested concentrations, the maximum rate of growth reached 0.497, 0.465, 0.421, 0.376, and 0. 312 mg/l while for Cu, these values reached 0.493, 0.461, 0.416, 0.335, and 0.294 mg/l, respectively. *Spirulina alga* has a high ability to bind metal ions from solution and bind with heavy metals due to the presence of functional groups that can bind with metal ions. The functional groups, especially carboxyl groups, amine, hydroxyl, and sulfate, are contained in the cytoplasm of the cell wall (Budi et al. 2020).

As pointed out previously, it appeared that the rate of growth was lessened in case of Zn and Cu than in case of Ni. The same trend was also recorded at the other concentrations of the three tested elements. This means that within the same element, the rate of growth was affected by the concentration of the element tested and the length of the culture period. Moreover, the results obtained proved that Cu ions whether at low or high concentrations were more toxic than both Ni and Zn ions.

Our results also go with the harmony of those explained by Soni et al. (2017) who observed that under stressed conditions, there was a significant change in the functional properties of *Spirulina*. Environmental stresses like high pH, light, salinity, temperature, nitrogen concentration, and different types of pollutants affect growth and nutrient productivity.

Ni^2+^ ions are known to be essential cofactors for four bacterial enzymes (Thauer 1983). Our results revealed that at lower concentrations of Ni (1.0 mg/l), the growth of *S. platensis* stimulated or enhanced, while the growth decreased and reached the lower values concentration at the maximum concentration of Ni (3.0 mg/l). Similar results were also obtained by Stratton and Corke (1979) who reported that high concentrations of Ni^2+^ were also toxic to *Chlorella* and *Anabaena* species. Furthermore, Stillwell and Holland (1977) proved that the rate of cell division in *Cricosphaera carterae* decreased progressively by increased nickel concentration.

The obtained results revealed also that *S. platensis* was able to display remarkable improvement at the worked lower concentration of nickel. In addition, Rai and Raizada (1986) observed maximum stimulation of growth in *Nostoc muscorum* at low concentrations of nickel. Besides, El-Mazally (2002) found that low concentrations of Ni^2+^ stimulated the growth of *Scenedesmus obliques* compared to control. Therefore, lower nickel values played a noteworthy role in the variation of biomass. The obtained results go in harmony with those obtained by Van Baalen and O’Donnell (1978) who found that the growth of one species of Cyanophyta has been reported to be dependent on nickel. Accumulation of nickel ions can be related to their intracellular gathering and fixation by *spirulina* biomass to decrease the toxicity (Zinicovscaia et al. 2016).

The results concerning the effect of different concentrations of Zn^2+^ ions on the growth of *S. platensis* cleared that Zn^2+^ ions are more toxic than Ni^2+^ ions on the growth of the alga. At lower concentrations (1.0 mg/l Zn^2+^), the rate of growth increased by 6.036% compared to control. The same result was observed by Cepoi et al. (2020) who indicated that *S. platensis* accumulates Zn in the biomass in amounts that exceeds the optimal amount determined for the growth. Zinc has great importance since it plays an important role in gene regulation and is involved in protein, nucleic acid, lipid, and carbohydrate metabolism. It is also considered an essential microelement for almost all classes of organisms (Ishimaru et al. 2011).

On the contrary, at concentrations 2.0, 2.5, and 3.0 mg/l Zn^2+^, the rate of growth decreased from the 10th day of culturing to the end of the experiment but the rate of decrease was more prominent at 3.0 mg/l than at 2.0 and 2.5 mg/l Zn^2+^. These results nearly coincide with those obtained by many authors: Fisher et al. (1981) reported an increase in growth of *Asteriorella japonica* responding to high copper and zinc levels, El-Naggar (1993) stated that high levels of zinc cause reduction in the growth of *Chlorella vulgaris* and *Scenedesmus bijuga*. Nalimova et al. (2005) reported that the increase in zinc concentrations (4.4 and 6.6 mg/l) reduced culture productivity of *Spirulia platensis* because zinc affects practically all physiological processes (i.e., membrane functioning, photosynthesis, cell division, and respiration). Similarly, Zinicovscaia et al. (2021) observed that *S. platensis* accumulated 2.5 mg Zn/g at zinc concentration in solution 2.5 mg/l.

The work presented here provides also that all tested concentrations of Cu^2+^ inhibited the growth of *S. platensis* measured as optical density but with different degrees. The lowest concentration (1.0 mg/l) accelerated the growth but increasing concentration of copper inhibited algal growth. Our results go in agreement with those obtained by Budi et al. (2020) who indicated that with the treatment of various levels of Cu, the extreme growth rate of *Spirulina plantesis* displays that the treatment by adding 1 ppm heavy metal is needed for increasing growth but the higher the concentration of Cu given, the lower the density of *Spirulina plantesis*. The importance of Cu^2+^ as an essential micro-nutrient and its effect in limiting algal growth and related metabolic activities was reported by many authors (Rai et al. 1991; Vymazal 1995; Budi et al. 2020).

Although Cu^2+^ is significant metal for living organisms, it can be lethal and cause the death of algal cells at high levels, and a decrease in growth and some important metabolites depended on increasing Cu concentration (Mohy El-Din 2017). Correspondingly, Stauber and Florence (1985) stated that this metal at levels greater than 5 mg/l reduced the growth of *N. closterium* by 50% below control. Copper was found more poisonous to the dinoflagellate *Prorocentrum micans* than the diatom (*Nitzschia closterium)* as reported by Carpene and Boni (1992). Furthermore, the poisonous copper effect on the growth of marine alga *Dunaliella tertiolecta* was obviously demonstrated in the cultures treated with the copper of 10 and 12 mg/l as recorded by Abalad et al. (1995).

Inhibition of algal growth by copper was reported in *Anabaena doliolum* (Rai et al. 1991 b), *Nostoc calcicola*, *Nostoc muscorum* (Pandy and Chatterjee 1999), *Chlorella vulgaris* (Brock 1969), and diatoms (Pistocchi et al. 2000). Furthermore, the results display that the inhibitory copper effect on the growth rate is higher pronounced than the other experienced metals (nickel and zinc). These findings are matching with several previously published results (Muwafq and Bernd 2006). Meenakshi (2007) observed high reduction in growth of *Spirulina paltensis* culture, and copper toxicity was higher than zinc by using 2 mg/l for both heavy metals. The toxicity of heavy metals may be remarked by changes in growth conditions and reduction in the growth of the tested microorganism.

**Fig. 9:**
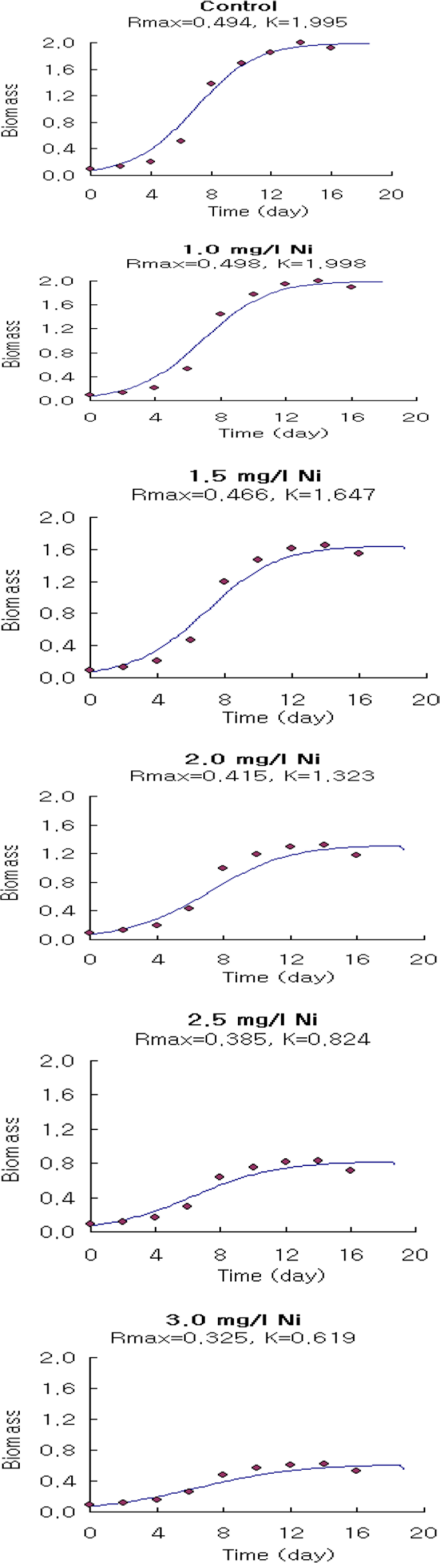
The output of the application of the logistic growth model fitting of *Spirulina platensis* cultured for 18 days under normal conditions (control) and under different concentrations of Ni^2+^

**Fig. 10:**
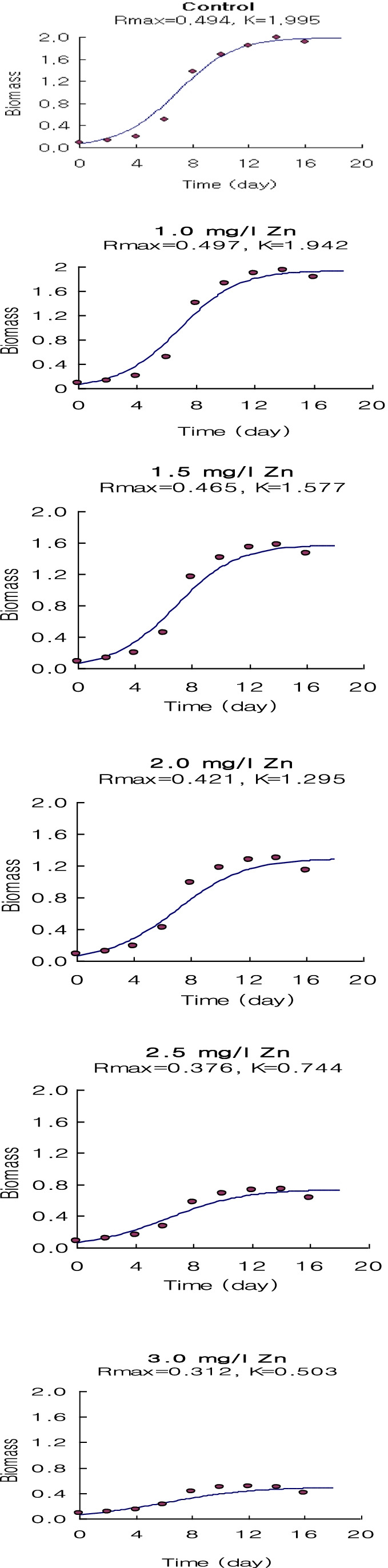
The output of the application of the logistic growth model fitting of *Spirulina platensis* cultured for 18 days under normal conditions (control) and under different concentrations of Zn^2+^

**Fig. 11:**
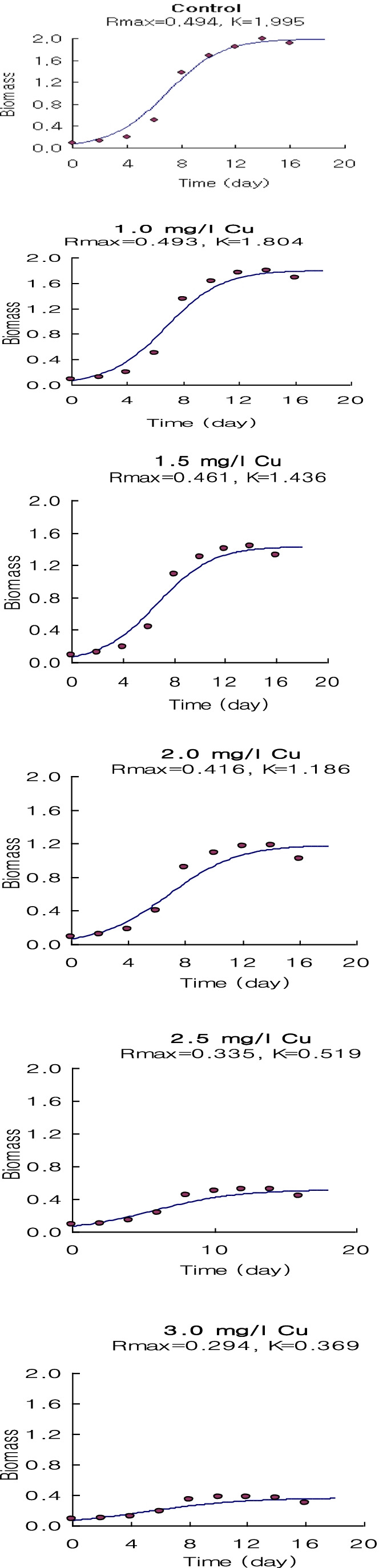
The output of the application of the logistic growth model fitting of *Spirulina platensis* cultured for 18 days under normal conditions (control) and under different concentrations of Cu^2+^

The logistic growth model proposed in this study can be used effectively to describe the results of algae inhibition growth tests (Figs. 9, 10, and 11). They do not have more factors than the routine analysis related to the logistic growth model which relates the population intrinsic growth rate to the heavy metal concentration. Wang et al. (2002) reported that the logistic growth model is better for in situ algal growth owing to the slow growth in the early phase and limited resources.

#### Effect of different concentrations of Ni^2+^, Zn^2^,^+^ and Cu.^2+^ ions on photosynthetic pigment fractions of Spirulina platensis

**Fig. 12:**
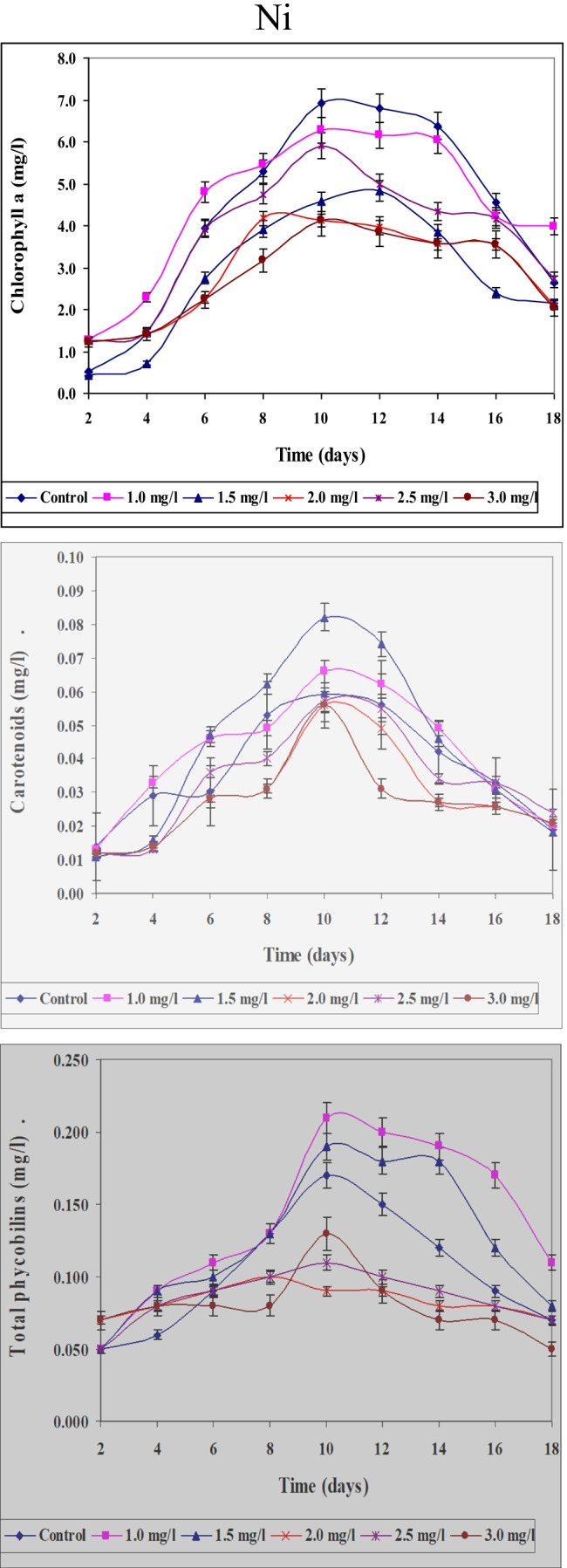
Effect of different Ni.^2+^ concentrations on chlorophyll a, carotenoids, and total phycobilins content (mg/l) of *Spirulina platensis* cultured for 18 days

**Fig. 13:**
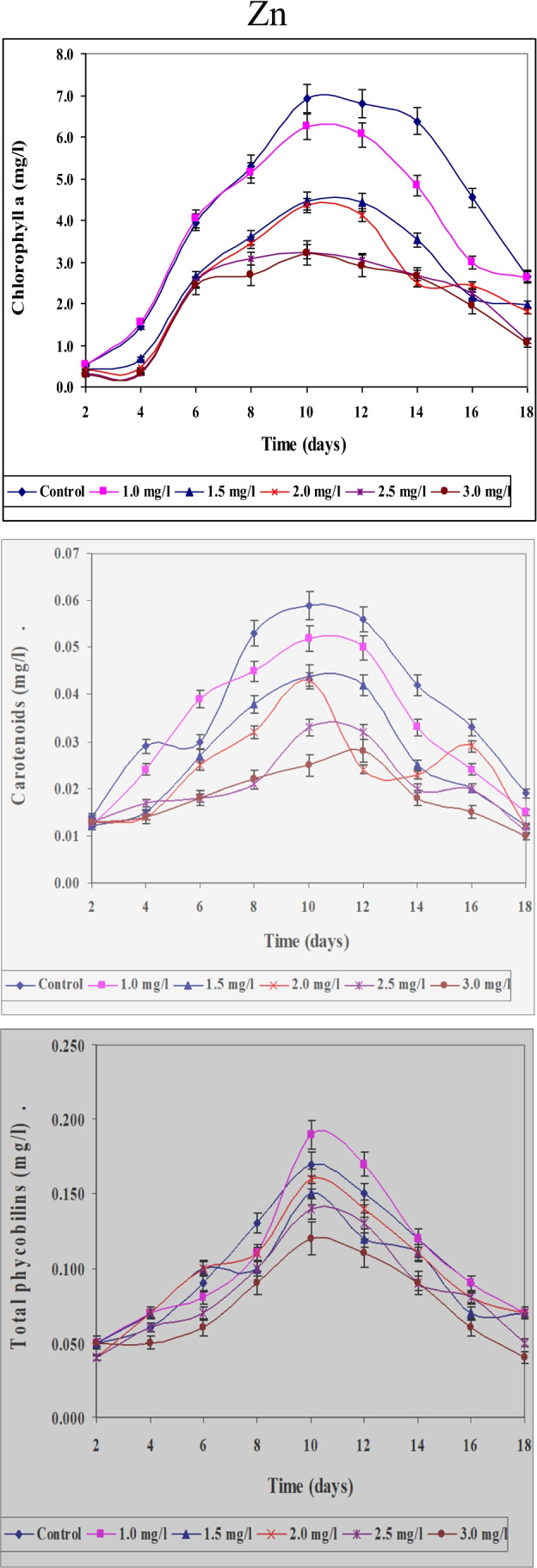
Effect of different Zn.^2+^ concentrations on chlorophyll a, carotenoids, and total phycobilins content (mg/l) of *Spirulina platensis* cultured for 18 days

**Fig. 14:**
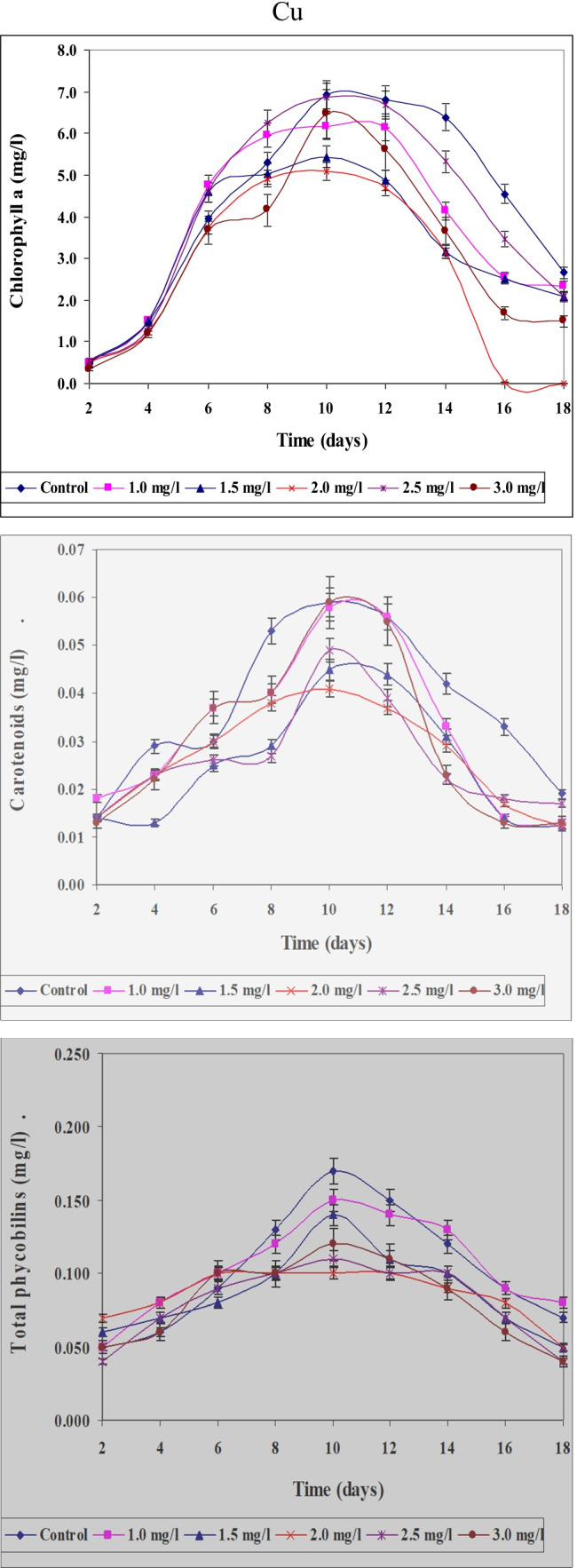
Effect of different Cu.^2+^ concentrations on chlorophyll a, carotenoids, and total phycobilins content (mg/l) of *Spirulina platensis* cultured for 18 days

Data concerning the effect of different concentrations of Ni, Zn, and Cu on content of chlorophyll a, carotenoids, and total phycobilins in *S. platensis* cultured for 18 days were represented in Figs. 12, 13, and 14. Anent data obtained for quantitative and for chlorophyll content as another parameter for growth determination concluded that the obtained results support also those obtained for optical density, i.e., maximum values of growth were recorded on the 10th day. This may support the idea that chlorophylls especially chlorophyll “a” could be used as a good parameter for growth determination. It must be mentioned here that chlorophyll content increased gradually with increasing the period of culturing till nearly the 10th day of the experiment. In this case, the results could be used only for constructing the growth curve of the organism. This means that in spite of the fact that optical density increased gradually with increasing the period of culturing, the content of chlorophyll decreased. This coincides with the results obtained by Markovits et al. (1993) who indicated that amount of chlorophyll decreased with the increase in culturing period together with the decrease of the available nutrients in the medium.

The present study indicates that application of low Ni^2+^ concentrations to cultures of *S. platensis* causes a significant increase in different pigment fractions (chlorophyll “a” phycobilins and carotenoids). On the other hand, higher Ni^2+^ concentrations suppressed the levels of these pigment fractions. These findings are in agreement with data obtained by Angadi and Mathad (1994) who found that chlorophylls were maximally inhibited at higher concentrations of Ni^2+^, but were stimulated at lower concentrations. Furthermore, similar results were obtained by El-Mazally (2002) for *Monoraphidium minutum* and *Scenedesmus obliques*.

These data proved that values of these parameters are differed according to concentrations of Zn and cleared also that Zn is more toxic than Ni ions. By increasing period of culturing, the toxicity of Zn was more prominent at higher concentrations 2.0, 2.5, and 3.0 mg/l than lower ones (1.0 and 1.5 mg/l). It is clear also that at higher concentrations of Zn, the decrease in the content of chlorophyll a and carotenoids was more prominent than that of phycobilins.

Chlorophyll “a” content followed nearly a similar pattern of change to that of growth responding to diverse concentrations of zinc. El-Naggar (1993) indicated that lower concentrations of zinc stimulate growth and increase the different pigment fractions of the green alga *Chlorella vulgaris* and *Scenedesmus bijuga.* However, higher concentrations of zinc suppressed the level of pigment fractions. Our results go with harmony with those obtained by Prassad and Prassad (1987) who found that low Zn^2+^ levels improved the total chlorophyll content in *Asterionella japonica*. High levels of heavy metals hinder the enzymes that are responsible for the chlorophyll synthesis. Zn^2+^ does not directly accelerate the reactive oxygen species formation owing to its redox inertness. Therefore, it exerted comparatively less stress on the investigated organism. Higher Zn^2+^ concentrations lead to decrease in cell division and total chlorophyll content (Afkar et al. 2010). The results cleared also that, the percentage of decrease in chlorophyll a content differed according to concentrations used and period of culturing. These results are in harmony with those obtained by Ghezelbash et al. (2008) who represented that reduction in chlorophyll content with reduced growth rate is due to decrease in photosynthetic rate.

Using different concentrations of Cu gave different values for the content of these pigment fractions (chlorophyll a, carotenoids, and total phycobilins). The results cleared also that Cu ions are more toxic to the content of pigment fractions of *S. platensis* than both Ni and Zn ions. In this study, the stimulatory effect of copper recorded during the first 2 days of culturing with lower concentrations can be accounted for either as a result of algal requirement of this element in metabolic processes or clarified by production of some organic compound which reduces metal toxicity. The poisonous effect of copper at higher concentrations may arise from the oxidative potential of copper (II) that causes reduction of chlorophyll (Muwafq and Bernd 2006). Our results are proven by those obtained by Budi et al. (2020) who recorded that 5 ppm Cu^2+^ is considered being a strong growth inhibitor of *Spirulina plantesis* due to inhibition of respiration for photosynthetic microalgae. Likewise, our results go matching with those obtained by Cepoi et al. (2020) who reported that copper toxicity for cyanobacteria can be explained by production of reactive oxygen species which leads to severe damage of lipids, proteins, and DNA and makes the substitution of Mg in chlorophyll. The inhibition in photosynthetic pigments accumulation responding to heavy metal stress may be also a magnitude of peroxidation of chloroplast membranes through the increased rate of reactive oxygen species production (Bajguz 2011).

Copper is an essential element for higher plants and algae (Breteler et al. 1984). Copper requirement could not be satisfied by any of other elements when ions of these elements were added singly to growth cultures lacking Cu^2+^ (Walker 1953). Copper is toxic to most organisms at high levels, therefore it is utilized in numerous molluscicides, fungicides, algicides, and marine antifouling compounds (Woolhouse 1983). Carotenoids and phycobilins content behaved the same way as in case of chlorophyll a. The total carotenoids at the lowest concentration of Zn^2+^ (1.0 mg/l) significantly increased gradually over control till the 8th day of culturing. At the other concentrations of Zn^2+^, the content of carotenoids and phycobilins decreased gradually till the end of the experiment. In the case of Cu^2+^, the decrease in the content of carotenoids and phycobilins was more prominent than in Zn^2+^. These results go in harmony with those obtained by El-Maghrabi (2002).

#### Assessing Idku Lake for growth of Spirulina platenses

Statistics of water quality parameters of Idku Lake are shown in Table 2. Most of the investigated parameters showed a noticeable fluctuation due to the impact of drainage water discharged into the lake from multiple sources. Neutral to weak alkaline water samples were reported since pH ranged from 7.30 to 8.60 with a mean of 7.86. High to low salinity water samples were observed due to the impact of saline seawater in the North and drainage freshwater in the South where TDS ranged from 1438 to 42,409 with a mean of 14,159 mg/l.

**Table 2:** Statistics of investigated water quality parameters to assess Idku Lake suitability

Statistics	pH	Turbidity NTU	TDS ppm	Nitrite ppm	Nitrate ppm	Phosphate ppm	Ammonia ppm	NDVI
Min	7.30	10.10	1438.00	0.06	2.75	3.55	0.03	− 0.35
Max	8.60	120.00	42,409.00	0.19	13.63	3.86	0.21	0.78
Mean	7.86	30.01	12,795.00	0.10	6.62	3.65	0.11	0.27
SD	0.38	29.73	14,159.89	0.04	3.18	0.10	0.07	0.20

Nutrients showed high levels at the eastern parts of the lake which might be due to the appearance of some agricultural drains nearby these sites. A great fluctuation is observed in the levels of turbidity among different sites which ranged from 10.1 to 120 with a mean 30.01 NTU. The high levels of turbidity are dominating the lake due to suspended particles and contaminants received with wastewater of drains. NDVI was employed to assess phytoplankton of the lake which showed the highest density in majority of the lake showing mean value 0.27 indicating the sparse density inside the lake.

**Fig. 15:**
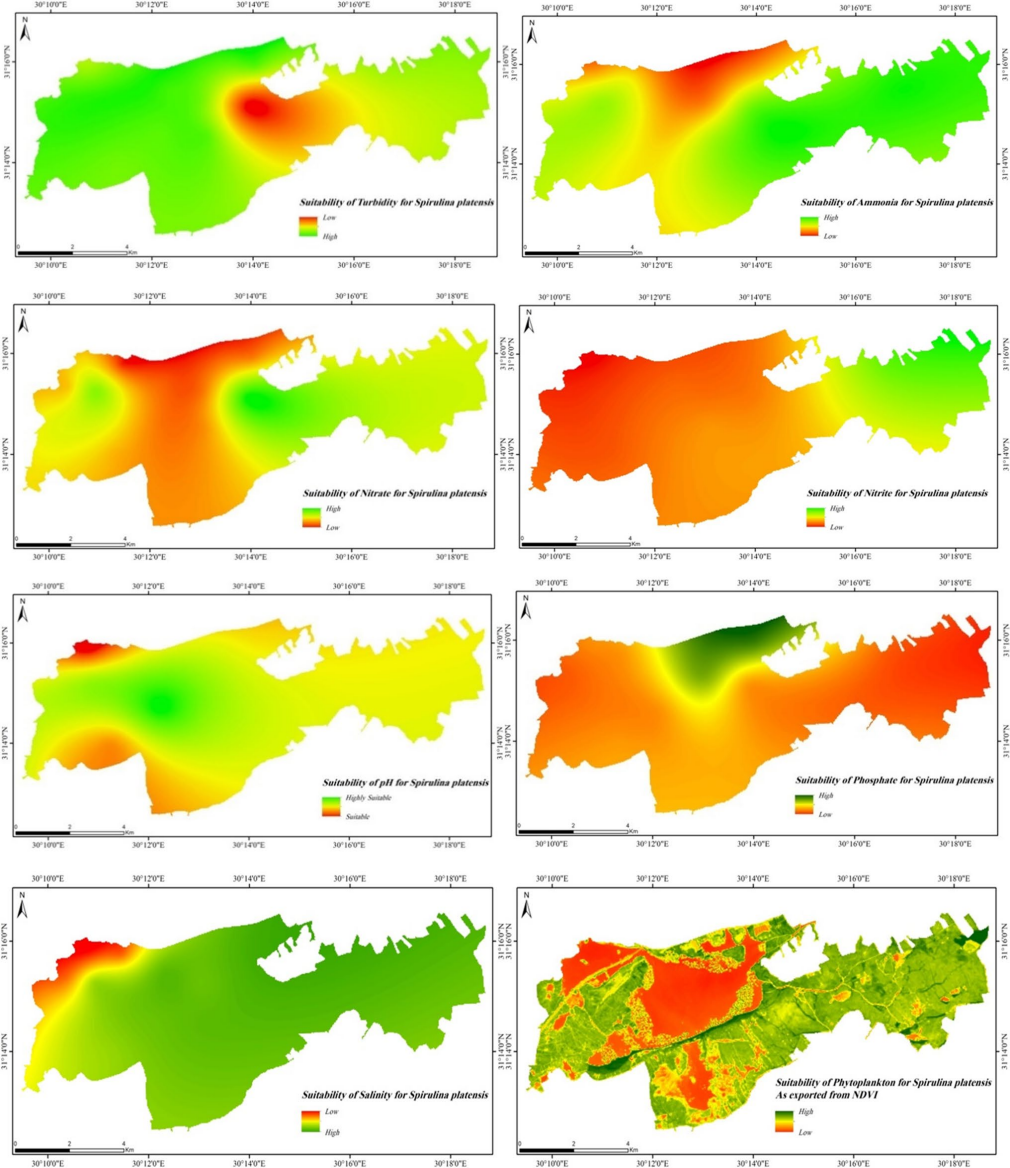
Suitability of different water quality parameters in Idku mg/l for *Spirulina platensis*

Based on the optimum conditions of the investigated parameters for the growth and spread of *Spirulina platensis*, the suitability spatial distribution maps were generated as shown in Fig. 15. Nitrate, nitrite, ammonia, salinity, and NDVI showed the same spatial patterns of suitability where eastern borders of the lake are more suitable than the western parts.

**Fig. 16:**
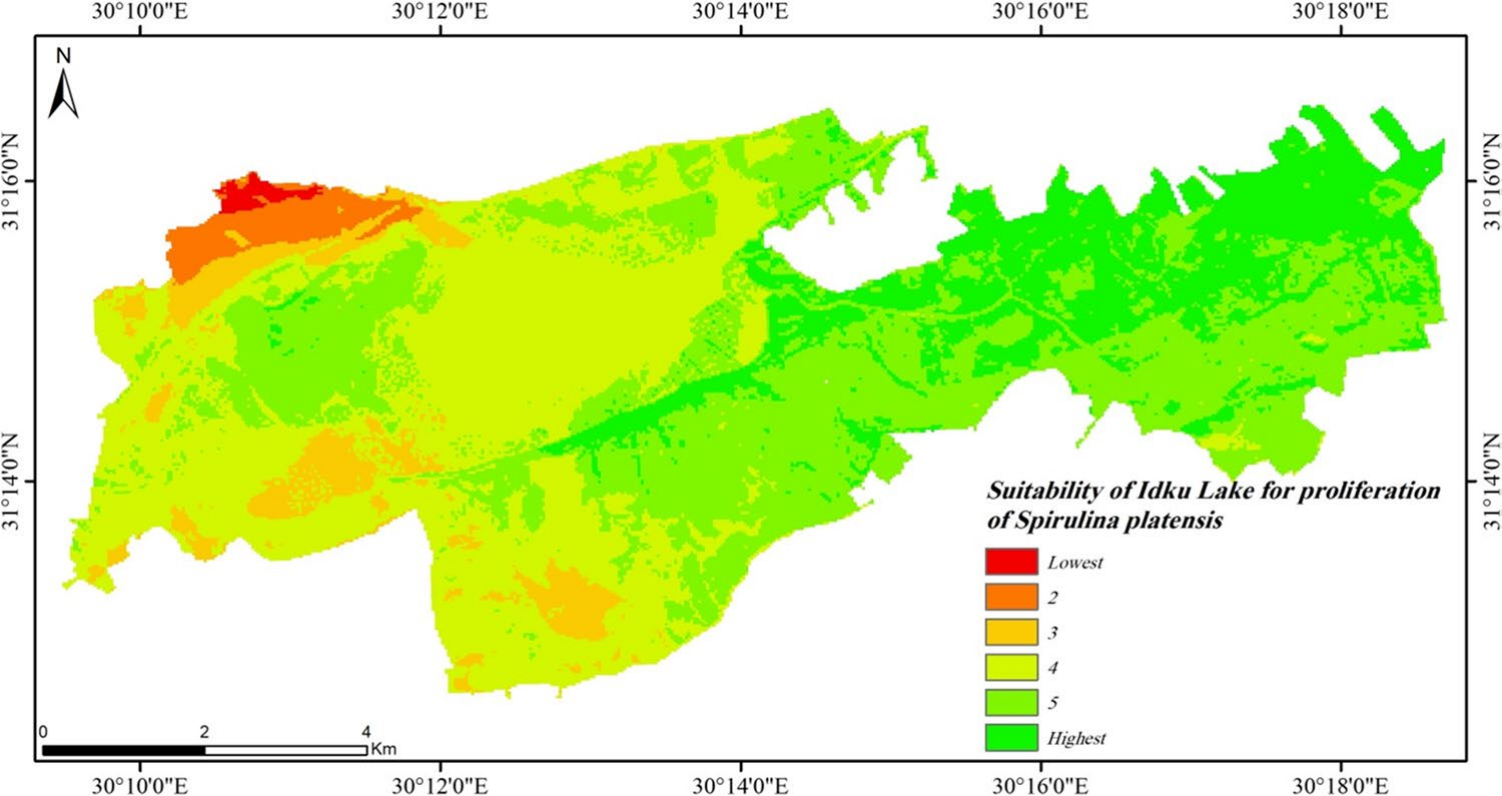
Suitability of water quality in Idku Lake for *Spirulina platensis*

The overall suitability map of *Spirulina platensis* in Idku Lake showed that the whole lake is suitable for the growth and proliferation except the northwestern corner due to the high salinity levels (Fig. 16). NDVI showed high levels in majority of the lake which indicates the appearance of different algal species. This means that the environmental conditions of the lake are promising for the spread of various algal species such as *Spirulina*, considering the potential impact on aquatic fauna.

## Conclusion

In Egypt, there is little information about the ambient concentrations of heavy metals. Algae are considered an excellent indicator for measuring different environmental pollutants. Risk assessment is a continually evolving process as important information on different contaminants, especially heavy metals. The health impacts involved and their occurrence in food are all factors that should be continuously measured and monitored. In this research, *Spirulina platensis* was selected for measuring the effect of different concentrations of three heavy metals (Ni, Zn, and Cu) on growth and photosynthetic pigment fractions due to the high importance of this type of algae. The suitability of Idku Lake was evaluated for proliferation of *Spirulina platensis* using geospatial techniques. Nitrate, nitrite, ammonia, salinity, and NDVI showed the same spatial patterns of suitability where the eastern borders of the lake are more suitable than the western parts. The results proved that the inhibitory effect of copper on the growth measured by optical density, growth rate, and mean growth rate was more pronounced than nickel and zinc under all the tested concentrations. In comparing toxicity of zinc and nickel, it was observed that Zn^2+^ ions are more toxic than Ni^2+^ ions. Lower concentrations of zinc and nickel stimulated growth and increased different pigment fractions (chlorophyll a, carotenoids, and total phycobilins content) while Cu^2+^ ions are more toxic to the content of pigment fractions than both Ni and Zn ions.

## Data Availability

All data generated or analyzed during this study are included and available in this article.
